# Importance of GPCR-Mediated Microglial Activation in Alzheimer’s Disease

**DOI:** 10.3389/fncel.2018.00258

**Published:** 2018-08-21

**Authors:** Md. Ezazul Haque, In-Su Kim, Md. Jakaria, Mahbuba Akther, Dong-Kug Choi

**Affiliations:** ^1^Department of Applied Life Science, Graduate School, Konkuk University, Chungju, South Korea; ^2^Department of Integrated Bioscience and Biotechnology, College of Biomedical and Health Science, Research Institute of Inflammatory Disease, Konkuk University, Chungju, South Korea

**Keywords:** GPCR, microglia, Alzheimer’s disease, amyloid beta, acetylcholine receptors, adrenergic receptors, dopamine receptors, purinergic receptors

## Abstract

Alzheimer’s disease (AD) is a progressive neurodegenerative disorder associated with impairment of cognition, memory deficits and behavioral abnormalities. Accumulation of amyloid beta (Aβ) is a characteristic hallmark of AD. Microglia express several GPCRs, which, upon activation by modulators, mediate microglial activation and polarization phenotype. This GPCR-mediated microglial activation has both protective and detrimental effects. Microglial GPCRs are involved in amyloid precursor protein (APP) cleavage and Aβ generation. In addition, microglial GPCRs are featured in the regulation of Aβ degradation and clearance through microglial phagocytosis and chemotaxis. Moreover, in response to Aβ binding on microglial Aβ receptors, they can trigger multiple inflammatory pathways. However, there is still a lack of insight into the mechanistic link between GPCR-mediated microglial activation and its pathological consequences in AD. Currently, the available drugs for the treatment of AD are mostly symptomatic and dominated by acetylcholinesterase inhibitors (AchEI). The selection of a specific microglial GPCR that is highly expressed in the AD brain and capable of modulating AD progression through Aβ generation, degradation and clearance will be a potential source of therapeutic intervention. Here, we have highlighted the expression and distribution of various GPCRs connected to microglial activation in the AD brain and their potential to serve as therapeutic targets of AD.

## Introduction

Microglia or resident macrophages of the central nervous system (CNS) originate from the embryonic yolk sac and are incorporated into the CNS during the earlier stages of development (Lannes et al., 2017). Microglia contribute to protection and maintenance of the CNS (Datta et al., 2018). Their role in the pathophysiology of many neurodegenerative disorders has been studied over the previous decades (Gentleman, 2013; Giunti et al., 2014; Salter and Stevens, 2017; Dukhinova et al., 2018), suggesting that microglia can rapidly change their phenotype to express different receptors according to stimuli generated by CNS damage or infection. Moreover, they can respond with both pro- or anti-inflammatory activity through direct migration (McHugh et al., 2010).

The progressive irreversible neurodegeneration disorder, Alzheimer’s disease (AD), is characterized by visible abnormal microscopic structures, such as deposition of the extracellular insoluble amyloid-β peptide (Aβ) in neuritic plaques and hyperphosphorylated tau protein in the neurofibrillary tangles (NFT) of the brain (Amihăesei et al., 2013; Luna et al., 2013; Puzzo et al., 2015). Accumulation of characteristic plaques and tangles in the brain consequently result in memory deficits and cognitive impairment in AD (Bloom, 2014). Aβ proteins are 37–43 amino acid containing peptides that are produced from precursor transmembrane Aβ precursor protein (β-APP) through sequential cleavage by β-and γ-secretase (Thinakaran and Koo, 2008). In the AD brain, Aβ_40_ and Aβ_42_ are abundantly found, whereas Aβ_42_ are closely related to AD pathogenesis (Crouch et al., 2008). Depending on Aβ aggregation and conformational changes, Aβ proteins exist in two distinct forms—soluble and fibrillar. Senile plaque, a characteristic hallmark of AD, is comprised of compact and dense insoluble Aβ fibrils and more soluble toxic oligomeric Aβ species (Shankar et al., 2008; Koffie et al., 2009). On the other hand, tau is a microtubule-associated protein, its breakdown, phosphorylation and changes in conformation have been implicated in the pathological progression of AD (Mondragón-Rodríguez et al., 2014; Yang et al., 2014). However, these characteristic biomarkers of AD can be detected a decade before the first symptoms of AD appear (Craig-Schapiro et al., 2009).

The link between microglia and AD was reported 30 years ago by McGeer et al. (1988) where the accumulation of microglia near the senile plaques in the AD brain was discussed (McGeer et al., 1988). In the AD brain, accumulation of this misfolded Aβ has been demonstrated to induce neuroinflammation by binding with several microglial innate immune receptors, including G-Protein-Coupled Receptors (GPCRs), which were postulated to induce an inflammatory cascade known as the “amyloid cascade-inflammatory hypothesis of AD” (Hardy and Higgins, 1992; Karran et al., 2011; McGeer and McGeer, 2013). This hypothesis is one of the most influential hypotheses surrounding AD pathogenesis to date. According to this hypothesis, the accumulation of Aβ and deficiency in its clearance is considered to be the root cause of AD pathogenesis and leads to extracellular senile plaque and tau-immunoreactive NFT formation, neurodegeneration and eventually dementia in AD patients (Hardy and Higgins, 1992). However, this hypothesis is challenged by many experts and it is believed that Aβ and tau are not the reason underlying AD pathogenesis, but merely its byproducts (McGeer and McGeer, 2013). Furthermore, it has been suspected that Aβ production triggers abnormal tau processing and thereby demands further study to determine a link between Aβ and tau protein (Zempel and Mandelkow, 2014). The role of microglia in Aβ formation, maintenance and clearance is highly investigated in the literature.

**Figure 1:**
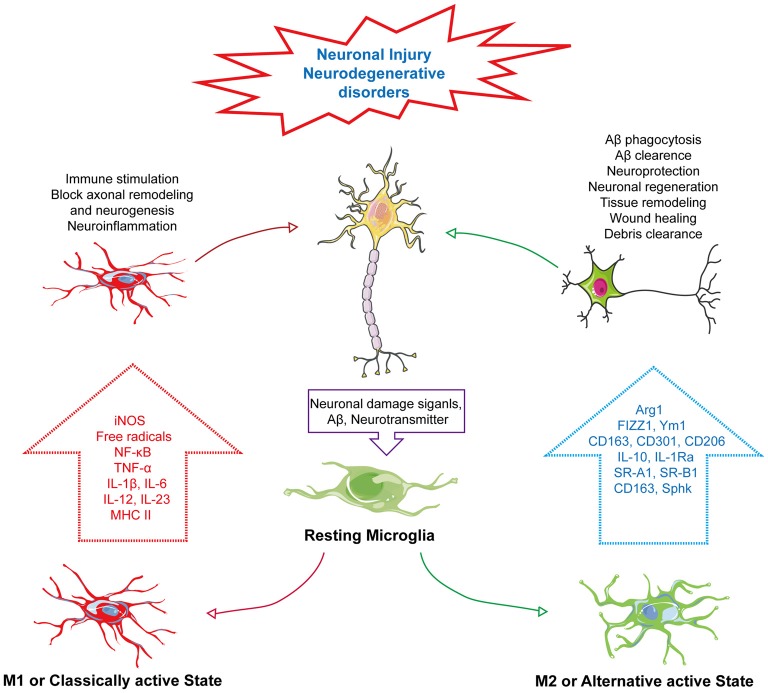
Phenotype change of microglia in neurodegenerative disorders. In response to neuronal damage, signals mediated from neurons, such as neurotransmitter or amyloid-β (Aβ), act as “on” or “off” signals for microglial activation. In response to “on” signals, there can be the alteration of its phenotype into two distinct states—M1: classically active state and M2: alternative active state. In the M1 state, microglia express iNOS and MHC II, activating the NF-κB pathway to produce several pro-inflammatory cytokines, such as tumor necrosis factor-α (TNF-α), IL-1β, IL-6, IL-12 and IL-23, and generate ROS and NO, which subsequently induce immune stimulation, neuroinflammation, block of axonal remodeling and prevent neurogenesis. Unlike the M1 active state, the M2 or alternative active state mediate neuroprotection through Aβ phagocytosis and clearance, modulate neuronal regeneration, and releases arginase 1 (Arg1) for tissue remodeling, wound healing and debris clearance through releasing M2 markers, Arg1, found in inflammatory zone 1 (FIZZ1), chitinase-like protein 1 (Ym1), triggering receptor also expressed on myeloid cells (TREM), CD163, CD301, CD206 and IL-1 receptor antagonist (IL-1Ra) and also expressing other markers, such as scavenger receptor class A1 (SR-A1), scavenger receptor class B1 (SR-B1) and sphingosine kinase (Sphk).

Based on the chemotactic effect of Aβ, microglia can accumulate near the core dense amyloid plaques. It has been postulated that, the chemotactic effect of Aβ plaques is related to its size. Therefore, microglial response can be changed in proportion to the size of Aβ plaque (Serrano-Pozo et al., 2013). Moreover, microglial phenotypic switch or change in activation state is closely associated with Aβ and plaques (Figure 1). It has been reported that smaller oligomers are more likely associated with Aβ induced microglial activation. Incubation of microglia with Aβ_1–40_ which produces smaller oligomers than Aβ_1–42_ showed marked increased in IL-6, NO release and tumor necrosis factor-α (TNF-α) expression (Takata et al., 2003). In another study primary microglia cells treated with freshly dissolved Aβ_1–40_ resulted in release of IL-1β and treatment with Aβ_1–42_ increased release of IL-1α and IFN-γ (Lindberg et al., 2005). Furthermore, the shifting of the microglial activation state from alternative to classical activation is linked with degradation and clearance of Aβ peptides through phagocytosis (Bard et al., 2000; Wilcock et al., 2003). This microglial activity in Aβ clearance and phagocytosis has a connection with GPCR-mediated signaling. GPCRs can modulate microglial activity through altering their response to many external and internal stimuli and control microglial morphology, chemotaxis and phagocytosis (Nagele et al., 2004; Nasu-Tada et al., 2005; Koizumi et al., 2007; Kim et al., 2011; Peng et al., 2015). P2Y_12_, a purinergic GPCR, activating microglia upon binding with extracellular nucleotides. Generally, expression of P2Y_12_ is high in resting microglia but it decreases upon microglial activation by adenosine diphosphate (ADP) or adenosine triphosphate (ATP). Moreover, microglia from P2Y_12_ knockout mice are unable to polarize and their chemotaxis is suppressed (Haynes et al., 2006; Ohsawa et al., 2007). Similarly, the orphan GPCR, G-Protein-Coupled Receptor 34 (GPR34), which belongs to the P2Y_12_-like group, is abundantly expressed on microglial membrane plays a vital role in microglial morphology, function and phagocytosis. Its deficiency in microglia has been reported to reduce phagocytosis in *Cryptococcus neoformans*-infected mice (Preissler et al., 2015).

The GPCRs were first identified in 1986—there are almost 800 members of GPCRs within the human genome, which makes it not only the largest superfamily of cell surface receptors but also the largest protein family in vertebrates (Dixon et al., 1986; Gloriam et al., 2007; Ghanemi, 2015). More than 370 of these GPCRs are of the non-sensory origin, involved in mood, cognition, pain and appetite. Almost 90% of these GPCRs can bind to a wide range of neurotransmitters and neuromodulators, like dopamine, norepinephrine and serotonin in the brain (Gainetdinov et al., 2004; Doze and Perez, 2012). Moreover, GPCRs in the brain can react to a wide variety of extracellular stimuli and perturbation of these GPCRs’ function can result in the pathogenesis of many neurodegenerative disorders, such as Parkinson’s disease and AD (Huang et al., 2017). For example, β-arrestin 2 has been found to increase Aβ production upon binding with two GPCRs, the orphan GPCR, G Protein-Coupled Receptor 3 (GPR3) and β_2_ adrenergic receptor, in both *in vitro* and *in vivo* models of AD (Jiang et al., 2013; Thathiah et al., 2013). Additionally, recent findings suggest GPR3 activity is linked to amyloidogenic proteolysis of amyloid-β precursor protein (APP) and its loss of activity is connected with memory improvement in AD transgenic (ADtg) mouse models (Huang et al., 2015). Neprilysin, a peptidase capable of breaking down Aβ in the brain, has been described to decrease its Aβ proteolytic activity by somatostatin hormone through GPCR-mediated signaling (Iwata et al., 2005). There are several microglial GPCRs, such as formyl peptide receptor 2 (FPR2) that bind to Aβ and mediates various inflammatory markers while also regulating Aβ degradation and clearance by cellular phagocytosis (Yu and Ye, 2015). As GPCRs are the most abundantly expressed receptors in the CNS and are connected to different downstream signaling pathways, potentially modulating Aβ degradation and proteolysis of APP through modulating α, β and γ-secretases, these unique features of GPCRs have made them the one of the most promising therapeutic targets for neurodegenerative disorders (Thathiah and De Strooper, 2011; Komatsu, 2015; Huang et al., 2017). Surprisingly, GPCRs are already the target of 475 (~34%) Food and Drug Administration (FDA)-approved drugs available today (Hauser et al., 2018). Within two decades, despite the advances of therapeutics for neurodegenerative disorders, the treatments of AD are mostly based on symptoms rather than its root cause or underlying pathology. In fact, the most popular and current treatments for AD to date are acetylcholinesterase inhibitors (AChEI) and N-Methyl-D-aspartate (NMDA) receptor antagonists (Mota et al., 2014; Gao et al., 2016).

Here, we would like to evaluate the functional and mechanistic relationship of GPCRs with microglia activation and importance of this phenomenon in AD. First, we would discuss the role of GPCRs in the activation of the microglia. Second, based on current reports and findings, we tried to expand the implication of GPCR-mediated microglial activation in this context to the pathophysiology of AD. Finally, we will focus on the therapeutic perspective of GPCRs as emerging drug targets for the development of novel therapeutic agents to treat AD.

## Microglial Activation and Neurodegeneration

Microglia, a motile phagocyte of our CNS. It is involved in neuronal cell defense from extremely harmful stimuli and capable of protecting cells from injury or death (Fu et al., 2014). On the other hand, microglia can change its activation to neurotoxic state. It’s because of the fact that microglia can switch their phenotype by a process called polarization (Hu et al., 2015). Polarization and changing of the phenotype are dependent on the types of CNS insults imposed on the brain and which type of mediator is produced in response (Hanisch and Kettenmann, 2007). It has been established for many decades that neuron cells are often the passive victims of microglia activation based on the accidental elimination of neurons when performing protective duties with respect to infection, damage or weakened selection pressures because of aging or neurodegenerative disorders (Brown and Vilalta, 2015). Microglia can shift to reactive states to deal with pathological contexts known as active states of microglia. However, many new studies have started to reveal the close intimacy of the microglia-neuron relationship regarding maintenance of the healthy state of the brain through bidirectional communication (Eyo and Wu, 2013). There is a probability that the cross-talk between these two cells can be achieved by neurotransmitters and their receiving receptors. We know that neurons can send different modulators to microglia requesting assistance to deal with pathological condition, though, on the other hand, microglia, upon receiving the signals, express diverse receptors to initiate feedback to maintain homeostasis (Peferoen et al., 2014; Wohleb, 2016). This wide array of signals triggers the activation of microglia through changing of microglia phenotype. These signals are basically of the “on” or “off” type; whereas the “on” signal is hypothesized to convert microglia to an activated state through a complex and multistage process, and through receptors, the “off” signal keeps microglia in the resting state (Liu et al., 2007). Activated microglia can migrate toward signals, proliferate and engulf injured cells (Kettenmann et al., 2011). There are two types of phenotypes of the active state of microglia—M1 and M2. The M1 phenotype is known as the classically activated state and M2 is referred to as an alternative active state that has other subpopulations (Mittelbronn, 2014). There many other subtypes of M2 states, like M2a, M2b, M2c and Mox, with each possessing unique characteristics with respect to different biological functions (Röszer, 2015). However, recent findings indicate that M1/M2 polarization is connected to several types of neurodegenerative diseases (Tang and Le, 2016). In response to neuronal injury, these two phenotypes have distinct characteristics. Upon the M1 activation state, microglia can release various proinflammatory cytokines, such as: IL-1β, TNF-α and IL-6, IL-12, IL-17 and IL-23; chemokines that include chemokine (C-C motif) ligand 2 (CCL2) and C-X-C motif chemokine 10 (CXCL10); express markers, cluster of differentiation 86 (CD86) and cluster of differentiation 16/32 (CD16/32); and free radicals through the NF-κB pathway that will adversely affect neuronal repair and regeneration (Mahad and Ransohoff, 2003; Kawanokuchi et al., 2006; Mosser and Edwards, 2008; Chhor et al., 2013; Davis et al., 2013; Franco and Fernández-Suárez, 2015; Subramaniam and Federoff, 2017; Che et al., 2018). Meanwhile, the M2 phenotype can act conversely by improving the brain’s repairing or regeneration processes by attenuating neuroinflammation. The M2 state of microglia is triggered by IL-4 or IL-13 that is activated by IL-10 or transforming growth factor-β (TGF-β; Ponomarev et al., 2007; Colton, 2009; Zhou et al., 2012; Subramaniam and Federoff, 2017). M2 increases phagocytosis activity and releases different markers, such as arginase 1 (Arg1) for tissue remodeling and wounding healing, chitinase-like protein 1 (Ym1), which prevents degradation of extracellular matrix components, Found in inflammatory zone 1 (FIZZ1), which blocks nerve growth factor-induced survival of the dorsal root ganglion neurons, CD163 hemoglobin-haptoglobin complex, triggering receptor also expressed on myeloid cells-2 (TREM2) for debris clearance, Dectin-1, leading to phagocytosis, CD301, for pathogen defense and is related to CD206, suppressor of cytokine signaling-3 (SOCS3), sphingosine kinase (Sphk), scavenger receptor class A1 (SR-A1), scavenger receptor class B1 (SR-B1), IL-1 receptor antagonist (IL-1Ra) and IL-10 (Figure 1; Holcomb et al., 2000; Willment et al., 2003; Raes et al., 2005; Morris, 2007; Neumann and Takahashi, 2007; Saba et al., 2009; Shechter et al., 2009; Chhor et al., 2013; Franco and Fernández-Suárez, 2015; Lisi et al., 2017; Subramaniam and Federoff, 2017).

Changes in microglial phenotype have a crucial impact on Aβ clearance during AD pathogenesis. In ADtg mice, it has been observed that acute intrahippocampal administration of lipopolysaccharide (LPS) in APP+PS1 transgenic mice reduces Aβ load (DiCarlo et al., 2001). In another study conducted on iNOS overexpression or iNOS mutant human β-amyloid precursor protein (hAPP) as well as human presenilin-1 (hPS1) expressing double transgenic mice to determine the role of iNOS, it was demonstrated that there are unpredictable reductions in Aβ levels as well as amyloid plaque formation with marked reduction in astrocytosis and microgliosis (Nathan et al., 2005). Changes in microglial phenotype were also reported in APP/PS1 mice. Shifting of the M2 state of microglia to the M1 state in the hippocampus of APP/PS1 mice was observed after 18 months of AD pathogenesis. Age-dependent shifting of microglial phenotype from alternative to classical was associated with increased downregulation of the M2 phenotypic marker Ym1 and TNF-α release (Jimenez et al., 2008). Similar results were found in an *in vitro* model that suggested that the shifting of the microglial state to the M1 phenotype ablated phagocytosis of Aβ. IL-4 or IL-10, two anti-inflammatory cytokines, are also involved in microglial polarization from M1 to M2 through affecting the Notch pathway, thereby increasing microglial Aβ phagocytosis (Koenigsknecht-Talboo and Landreth, 2005; Michelucci et al., 2009). In all, the aforementioned findings indicate a clear connection between microglial activation and its balance between different phenotypes playing a significant role in neurodegenerative disorders.

## Microglial GPCR-Mediated Effect on Aβ in AD

Since the identification of microglia by Rio-Hortega (1932), microglia have become the centerpiece of a variety of infectious, inflammatory and neurodegenerative disorders of the CNS based on its dual characteristic in response to external stimuli to the brain (Rio-Hortega, 1932; Luo and Chen, 2012). In AD, Aβ can directly bind to microglia and activate them. Numerous studies have reported that GPCRs can directly influence the amyloid cascade by exploiting α, β, γ-secretase, APP proteolysis and Aβ degradation (Thathiah and De Strooper, 2011). On the other hand, microglia are also capable of clearance of Aβ by receptor-mediated phagocytosis (Table 1, Ries and Sastre, 2016; Clayton et al., 2017). Microglial expression of different membrane receptors is intended to respond to the disturbance in neuronal damage. GPCRs expressed by microglia have been shown to regulate distinct components of their activation process, including cell proliferation, migration and differentiation into M1 or M2 phenotypes (Fung et al., 2015). Among these diverse receptors types, GPCRs play a pivotal role in the modulation of various components of microglial activation (Table 1). With this, the involvement of GPCRs and its subtypes in neurodegenerative disorders have been implicated in many studies (Heng et al., 2013; Guerram et al., 2016). Moreover, there are many other uncharacterized GPCR subtypes that are featured in microglial activation and need to be investigated for their pharmacological and molecular activity in AD (Stella, 2010).

**Table 1:** Reported microglial-G protein-coupled receptors (GPCRs) and their role in Alzheimer’s disease (AD).

Microglial GPCRs	Sub-types	Endogenous modulators	Synthetic modulators	Mechanism	Role in AD	Reference
nAChR	α7 nAChRs	Aβ, Choline, Kynurenic acid	Galantamine	↑NO, ↑TNF-α expression and ↑IL-6 activation induces ↑Ca^2+^ influx, ↑Calmodulin-CaMKII pathway	↑Aβ clearance through microglia phagocytosis	Takata et al. (2010) and Steiner et al. (2014)
mAchRs	M1	Dopamine	Carbachol, Aβ_(40, 42 and 25)_, AF267, Dicyclomine, C-2 ceramide	↑PKC-α, ↑PKC-γ and/or ↑CREB, ↑pMAPK	↑α-secretase, ↑sAPP release and P3, ↑Aβ generation	Buxbaum et al. (1992); Hung et al. (1993); Farber et al. (1995); Caccamo et al. (2006); Joseph et al. (2006) and Joseph et al. (2007)
Adenosine	A_1_	ATP, Adenosine	Caffeine, DPCPX, SCH58261	↓Ca^2+^ influx, ↑Cyclic nucleotide signaling, ↑p21 Ras activation, ↑ERK1/2 phosphorylation	↓Microglial activation, ↑neuronal damage ↑phosphorylation and translocation of tau, ↑Aβ toxicity	Schubert et al. (2000); Angulo et al. (2003); Giunta et al. (2014); and Luongo et al. (2014)
	A_2A_	Adenosine	Caffeine, DPCPX, SCH58261	↑Cyclic nucleotide signaling	↑Neuronal damage, ↑Aβ toxicity	Schubert et al. (2000) and Giunta et al. (2014)
	A_2B_	Adenosine	MRS1754, BAY60-6583	↑IL-6 and ↑IL-10, ↑p38 MAPK, ↑pCREB	↓Microglia activation	Koscsó et al. (2012) and Merighi et al. (2017)
	A_3_	Adenosine	Cl-IB-MECA, and MRS1523	↓PI3 kinase/Akt, ↓NF-κB and ↓TNF-α	↓Microglia activation	Hammarberg et al. (2003) and Lee et al. (2006)
Purinergic	P2Y_2_	ATP and UTP		↑Nox	↑Aβ degradation and clearance	Kim et al. (2012); Mead et al. (2012) and Ajit et al. (2014)
	P2Y_4_	ATP		↑Nox, the ↑PI 3-kinases/Akt cascade	↑Microglial uptake of Aβ	Mead et al. (2012) and Li et al. (2013)
	P2Y_6_	UDP	MRS2578	↑NFATc1, ↑c2, ↑CCL2 and CCL3 production	↑Microglial chemotaxis, ↑Microglial phagocytosis	Koizumi et al. (2007) and Kim et al. (2011)
	P2Y_12_	ADP		↑cAMP-dependent PKA	↑Microbial chemotaxis	Nasu-Tada et al. (2005)
	P2Y_13_	ADP		↑cAMP-dependent PKA	↑Microbial chemotaxis	Nasu-Tada et al. (2005)
mGluRs	**Group I** (mGluR1 and mGluR5)		Triptolide (T10), CHPG, MTEP and VU0360172	↓iNOS, TNF-α, and IL-1β and IL-6 and MAPKs pathway, ↑Shedding of the microvesicle from microglia, ↑Microglia-induced astrocyte	↓Microglial-mediated neurotoxicity, modulate microglia-neuron communication	Beneventano et al. (2017) and Huang et al. (2018)
	**Group II** (mGluR2 and mGluR3)		LYY37926, (RS)-α-methyl-4-sulphonophenylglycine	mGluR2: ↑TNF-α release and caspase-3 activation and FasL expression, mGluR3:↑BDNF	mGluR2: ↑Microglial neurotoxicity, ↑sAPPα, ↑Non-amyloidogenic cleavage of APP. mGluR3: ↑sAPPα and ↓Amyloid level. Switching of microglial phenotype to neurotoxic phenotype	Kingham et al. (1999); Taylor et al. (2002); Taylor et al. (2003); Durand et al. (2014) and Durand et al. (2017)
	**Group III** (mGluR4, mGluR6, mGluR7 and mGluR8)		(L)-2-amino-4-phosphono-butyric acid (L-AP-4), (R, S)-phosphonophenylglycine (RS-PPG)	↓Microglial glutamate release, ↓Excitotoxicity, ↑Astrocytic glutamate	↓Microglia-mediated neurotoxicity	Taylor et al. (2003)
Adrenergic	α_2A_		Nonspecific Atipamezole, BRL-44408 and Dexmedetomidine (DEX)	↓TLR4 overexpression, ↓IL-4, ↓Arg-1, ↓Resistin-like α (Retnla/Fizz1), and ↓Chitinase 3-like 3 (Chi3l3/Ym1) expression	↓Cognitive impairment, ↓Polarization of microglia to M1	Yamanaka et al. (2017) and Zhang et al. (2017)
	β_1_	.	Xamoterol and STD-101-D1	↓Iba1 and GFAP, ↓(Iba1, CD74, CD14 and TGFβ), ↓TNF-α	↓Microgliosis	Ni et al. (2006); Yu et al. (2011) and Ardestani et al. (2017)
	β_2_		Isoproterenol		↑α secretase activity, ↑Aβ level	Ni et al. (2006)
FPRL1/2		Aβ_42_	Aβ_42_, Annexin A1 (ANXA1), Humanin, palmitoyl-cys[(RS)-2, 3-di(palmitoyloxy)-propyl]-Ala-Gly-OH (PamCA), and muramyl dipeptide (MDP)	↑TNF-α and ↑MAPK p38	↑Microglial chemotaxis, ↓Aβ level	Cui et al. (2002); Ying et al. (2004); Iribarren et al. (2005); and Ries et al. (2016)
CMKLR1		Aβ_42_		↑ERK1/2, PKA, and Akt	↑Processing and clearance of Aβ_42_	Peng et al. (2015)
Chemokine receptors	CCR5 CX_3_CR1	CCL2 CCL3 CCL4 CXCL8		NA	Associated with amyloid deposits, ↓Microglial neurotoxicity, ↓γ-secretase activity	Xia et al. (1998); Bakshi et al. (2008) and Hickman and El Khoury (2010)
Cannabinoid receptors	CB1	Endocannabinoids	Tetrahydrocannabinol (THC), Agonist: HU-210, WIN55, 212–2, and JWH-133	↓NADPH oxidase reactive oxygen species, ↓IL-1β and TNF-α and NO	CB1 expression decreased as AD progresses	Howlett et al. (1986); Glass and Felder (1997); Ramírez et al. (2005); and Manuel et al. (2014)
	CB2	Endocannabinoids	Tetrahydrocannabinol (THC), Agonist: AM1241, HU-210, WIN55, 212–2, and JWH-133 Antagonist: AM630	↓IL-6, TNF-α and free radical production	↓Aβ-induced microglial activity	Howlett et al. (1986); Glass and Felder (1997); Facchinetti et al. (2003); Walter et al. (2003); Carrier et al. (2004); Ramírez et al. (2005); Eljaschewitsch et al. (2006); Bisogno and Di Marzo (2010); and Ma et al. (2015)
	GPR55	Lysophophatidylinositol (LPI)	Abnormal-cannabidiol (Abn-CBD), Antagonist: CID16020046	↑ERK phosphorylation	Involved in spatial learning and memory, motor function, memory formation and neuroinflammation	Brosnan and Brosnan (2013) and Stojanovic et al. (2014).
Orphan GPCRs	GPR18		N-arachidonoyl glycine (NAGly)	↑MAPK activation	↑Microglial migration to neuronal damage	McHugh et al. (2010)

*Up arrow denote increase and the down arrow denotes decrease*.

### Purinergic Receptors

ATP acts as an extracellular signal taking part in neuromodulation and neurotransmission through activating purinergic receptors (P2) and mediating purinergic signaling in the CNS. P2 receptors are categorized into two receptor families—ATP-gated ion channels (P2X) and GPCRs (P2Y; Burnstock, 2017). P2X receptors are further subdivided into seven subtypes: P2X_1_, P2X_2_, P2X_3_, P2X_4_, P2X_5_, P2X_6_ and P2X_7_ receptors as well as P27. GPCRs, P2Y receptors, are further subdivided into eight subtypes, comprising five G_q_-coupled receptors—P2Y_1_, P2Y_2_, P2Y_4_, P2Y_6_, and P2Y_11_—and three G_i_-coupled receptors—P2Y_12_, P2Y_13_ and P2Y_14_ (Peterson et al., 2010). A handful of evidence indicates that ATP release is linked to Aβ generation. Numerous studies have also reported the involvement of P2Y receptors in Aβ generation and depletion and neuroinflammation mediated by Aβ toxicity in AD (Erb et al., 2015). Extracellular ATP generated from damaged tissue and astrocytes tends to attract microglial response to brain injury-activating P2Y receptors and connexin channels (Davalos et al., 2005). The microglial P2Y_2_ receptor has been implicated to enhance Aβ degradation via microglial activation. To find a relationship between Aβ clearance and P2Y_2_ receptor a study was carried out in an AD mice model using TgCRND8 transgenic mice with homozygous or heterozygous P2Y_2_ receptor deletion. The study demonstrated that APP mice with homozygous deletion of P2Y_2_ receptors showed comparatively more Aβ clearance and a greater survival rate with mild expression of the microglial marker, CD11b than heterozygous P2Y_2_ receptor deletion (Ajit et al., 2014). Another study in P2Y_2_ knockout mice confirmed the involvement of microglial P2Y_2_ receptors in Aβ clearance. In that investigation, primary microglia cells treated with ATP and uridine diphosphate (UDP) exhibited a marked increase in Aβ_1–42_ uptake whereas it shows no increase in primary cells isolated from P2Y_2_R knock-out mice. The ablation of Aβ_1–42_ uptake was speculated to be based on the inhibition of the α_V_ integrin-, Src- and Rac-mediated P2Y_2_R signaling pathways (Kim et al., 2012).

In addition, it has been claimed that microglial P2Y_4_R is linked to previously unreported microglial pinocytosis mediated uptake of Aβ. The pinocytic effect of microglia was modulated by the phosphatidylinositol 3-kinase/Akt cascade in the absence of ATP (Li et al., 2013). Exceptionally, the P2Y_4_ receptor in humans is seen to be antagonized by ATP in contrast to rat P2Y_4_ receptors. Stability of P2Y_4_ receptors in both human and rat astrocytoma cells were measured by intracellular Ca^2+^ levels. Although these same receptors in two different species share 83% identical amino acid sequences, they have contrasting pharmacological activity (Kennedy et al., 2000). Depending on the expression of receptors microglia superoxide production through Nox activation can change microglial migration to either neurotoxic or neuroprotective phenotype. Nox can affect the microglial response to external stimuli depending on its agonist. However, P2Y_(2/4)_ receptors are integral to the activation of Nox and the neurotoxic profile of microglia in response to changes in neurodegenerative stimuli (Mead et al., 2012). When hippocampal neurons are damaged, they release UDP. In response to UDP activating glial cells, which rapidly produce CCL2 and CCL3, as a result, chemokine levels rise by activation of the P2Y_6_ receptor. In addition, UDP also activates NFATc1 and c2, two calcium-activated transcription factors. In two different studies, it was demonstrated that attenuating the P2Y_6_ receptor-mediated pathway by inhibiting phospholipase C and calcium or administering P2Y_6_-specific antagonists (MRS2578) led to a significant decrease in chemokine expression (Kim et al., 2011). Moreover, kainic acid administration elevated the release of UDP with markedly enhanced P2Y_6_ receptor activation, which subsequently led to neuronal cell death both *in vivo* and *in vitro*, which suggests that UDP may act as a sensor for microglia-mediated phagocytosis in response to neuronal damage in neurodegenerative disorders (Koizumi et al., 2007). Furthermore, the role of G_i_-coupled receptors—P2Y_12_ and P2Y_13_ mediating microglial chemotaxis by ADP and ATP has been reported in many studies (Honda et al., 2001; Haynes et al., 2006). According to Nasu-Tada et al. (2005), ADP induces microbial chemotaxis in the presence of fibronectin and in a β_1_ integrin-dependent manner, whereas cAMP-dependent PKA positively regulates β_1_ integrin-induced microglial proliferation, which was reduced by purinergic signals from P2Y_12/13_ (Nasu-Tada et al., 2005).

### Adenosine Receptors

Adenosine is a neurotransmitter expressed in different cells of the CNS, including glia, and plays a pivotal role in the neuronal excitation, synaptic transmission and neuronal excitability (Ribeiro and Sebastiao, 2010). There are four subtypes of adenosine receptors: A_1_, A_2A_, A_2B_ and A_3_ receptors, which belong to purinergic GPCR family. The imbalance of adenosine receptor subtypes is associated with cognitive functional issues in AD (Yan et al., 2014). In the human brain, high expression of A_1_ and A_2A_ receptors is seen in the frontal cortex (Albasanz et al., 2008). The A_2A_ receptor is generally expressed in a striatal neuron in healthy brains, but in AD patients, it is expressed in the glial cells in the hippocampus and cerebral cortex region (Angulo et al., 2003). Microglia treated with ATP showed upregulation of A_1_ receptors and upon selective modulation of A_1_, suppressed microglial activation through decreasing Ca^2+^ influx (Luongo et al., 2014). In addition, the rise of extracellular adenosine levels have been reported to mediate both A_1_ and A_2A_ receptor activation and cyclic nucleotide signaling to counteract glial cell-mediated neuronal damage in AD (Schubert et al., 2000). A_1_ receptors are abundantly present in the CA1 region of the hippocampus of the normal human brain. Activation of A_1_ receptors subsequently causes p21 Ras activation and ERK1/2 phosphorylation, which results in phosphorylation and translocation of tau in an ERK-dependent manner (Angulo et al., 2003). Suppressing the activity of these (A_1_ and A_2A_) has been found to prevent Aβ toxicity *in vitro* (Giunta et al., 2014). A_3_ receptor activation in the murine microglial cell has been reported. Activation of the A_3_ receptor by adenosine and Cl-IB-MECA, a selective adenosine A_3_ receptor agonist, tend to inhibit PI3 kinase/Akt and NF-κB and eventually production of TNF-α in BV-2 microglia cells. On the other hand, MRS1523, a selective A_3_ receptor antagonist, reverses such an effect (Hammarberg et al., 2003; Lee et al., 2006). In a microglia-mediated neuroinflammation model, A_2B_ receptors have been reported to stimulate IL-6 and IL-10 production through the p38 mitogen-activated protein kinase (MAPK) pathway and phosphorylation of cAMP response element-binding protein (CREB; Koscsó et al., 2012; Merighi et al., 2017).

The capability of adenosine to regulate microglial activation proliferation and chemotaxis through A_2A_ receptors has made the A_2A_ receptor an anticipated therapeutic target for treating the diseases that are linked to microglial activation (Santiago et al., 2014). In addition, 2’,3’-cAMP and its metabolites (3’-AMP, 2’-AMP and adenosine), precursors of adenosine involved in the extracellular adenosine pathway, have been reported to suppress different cytokines and chemokines, like TNF-α and CXCL10, being released from the activated microglia (Newell et al., 2015). As cytokines or chemokines are found predominantly in the transition of the microglial phenotype from M1 to M2, the role of A_2A_ receptors should receive more attention (Franco and Fernández-Suárez, 2015). Spatial recognition memory improvement has been observed in mice lacking A_2A_R while the opposite was seen in transgenic mice overexpressing A_2A_ receptors (Wang et al., 2006; Giménez-Llort et al., 2007). Preladenant, a selective antagonist of adenosine A_2A_ receptors, has been reported to restore the microglial response towards cellular damage and plaque-associated microglial function in AD (Gyoneva et al., 2016). Caffeine, an antagonist of both A_1_ and A_2A_ receptors, has been widely studied against Aβ-induced neurotoxicity and cognitive impairments in both *in vivo* and *in vitro* models of AD and exhibited much potential in the management of cognitive dysfunction (Querfurth et al., 1997; Arendash et al., 2006; Dall’Igna et al., 2007). It seems clear that A_2A_ receptor plays a vital role in the activation of microglia in AD, therefore designing a selective antagonist of this receptor will prove a breakthrough in AD treatment (Abbracchio and Cattabeni, 1999; Nobre et al., 2010).

### Metabotropic Glutamate Receptors

Glutamate is abundantly found throughout the CNS—it is an excitatory neurotransmitter that features prominently in maintaining communication between neurons, microglia, astroglia and oligodendrocytes (Brosnan and Brosnan, 2013; Stojanovic et al., 2014). Imbalance of glutamate signaling plays an important role in AD pathogenesis (Lan et al., 2014). There are two types of glutamate receptors (GluRs)—ionotropic and metabotropic, and microglia express both types of receptors (Noda, 2016). Based on sequence homology, coupling with G proteins and ligands, metabotropic glutamate receptors (mGluRs) are further divided into three subgroups: Group I consists of mGlu1 and mGlu5 receptors; Group II consists of mGlu2 and mGlu3 receptors; and Group III consists of mGlu4, mGlu6, mGlu7 and mGlu8 receptors (Niswender and Conn, 2010). Recently, the function of mGlu5, a Group I mGluR in microglia activation, has been investigated (Xue et al., 2014; Zhang et al., 2015). In an LPS-induced neuroinflammation model, Triptolide (T10), a potent inhibitor of microglia activation has been reported to attenuate inflammation and show immunosuppression through mGlu5 receptor upregulation. In both BV-2 microglia and primary microglia cells, blocking or knocking down of mGlu5 receptors abolishes the anti-inflammatory effect of T10. T10 also blocks the LPS-induced expression of iNOS, TNF-α, IL-1β and IL-6 through modulating the MAPK pathway (Huang et al., 2018). These findings suggest the role of mGlu5 in neuroprotection against microglial activation-mediated neurotoxicity. Another striking finding illustrates mGlu5 receptors’ role in microglial communication with neurons through microglial macrovesicles (MV) shed. MV shedding from BV-2 microglia cells was produced upon activation of purinergic receptor P2X7 by benzoyl-ATP, which is elevated by treatment upon CHPG, a mGlu5 receptor agonist and ablated by LPS treatment. The results of this study demonstrated that MV produced from CHPG-treated BV-2 microglia significantly rose rotenone-induced neurotoxicity in SHSY5Y cells. An increased level of miR146a, a miRNA only found in MV produced from CHPG-treated BV-2 cells, has been postulated to be responsible for mGlu5-mediated neurotoxicity, which proves the involvement of microglial mGlu5 receptors in neuronal death (Beneventano et al., 2017). However, further research is required to clarify the underlying mechanism in mGlu5 receptor-mediated MV shedding and its possible implication in neurotoxicity. On the other hand, group II mGluRs are involved in the switching of microglial phenotypes to a neurotoxic phenotype upon activation by Chromogranin A (CGA). In contrast, a specific antagonist, (RS)-α-methyl-4-sulphonophenylglycine, of these receptors diminishes microglial activation and neurotoxicity (Kingham et al., 1999; Taylor et al., 2002). The neurotoxic effect of mGluR2 upon activation by L-2-amino-4-phosphono-butyric acid, a specific group III receptor agonist, is associated with TNF-α release and caspase-3 activation, which was potentiated by a death receptor ligand, Fas (Taylor et al., 2003). Durand et al. (2014) reported the link between mGluR3-mediated microglial activation in the pathogenesis of AD. Their work provided evidence that activation of mGluR3 by its agonist, LYY379268, increased soluble amyloid Precursor Protein-α (sAPPα) by facilitating the non-amyloidogenic cleavage of APP (Durand et al., 2014). This finding was further validated by their very recent investigation proving mGluR3 activation in glial cells has a dual function, such as increasing BDNF and sAPPα levels and reducing amyloid from cells by phagocytosis (Durand et al., 2017). Altogether, the evidence suggests the multifunctional role of mGluR2- and mGluR3-mediated microglial phagocytosis, switching of microglial phenotype, and non-amyloidogenic cleavage of APP. These findings indicate their potential value as therapeutic targets for AD. Besides the well-documented role of microglial group II mGluRs in AD, very little is knowabout the involvement of microglial group III mGluRs in AD. In neurodegenerative diseases the activation of microglial group III mGluRs can modulate the release of stable neurotoxins from microglia and protects neurons from microglial neurotoxicity (Taylor et al., 2003). In addition, activation of group III mGluRs can encourage microglia to adopt neurotrophic phenotype. Microglia adopt neurotrophic phenotypes through decreasing the glutamate release and suppressing excitotoxicity as a result of astrocytic glutamate release being elevated. (Williams and Dexter, 2014). Two agonists of group III mGlu receptors—(L)-2-amino-4-phosphono-butyric acid (L-AP-4) and (R,S)-phosphonophenylglycine (RS-PPG)—showed a reduction of microglial neurotoxicity induced by LPS, CGA or Aβ_(25–35)_ (Taylor et al., 2003). Therefore, selectively targeting microglial group III mGluRs may prove beneficial to understand microglial neurotoxicity in AD and to design effective therapeutic agents.

### Adrenergic Receptors

Noradrenaline neurotransmitters has been reported to influence microglial activation in response to pathogenic conditions (Gyoneva and Traynelis, 2013). They operate by an adrenergic receptor which is divided into two groups, namely α and β adrenergic receptors, and further subdivided into several subtypes, including α_1_, α_2_ (subtypes α_2A_-, α_2B_- and α_2C_), β_1_, β_2_ and β_3_ (Ciccarelli et al., 2017). This distinct family of GPCRs couples with G_s_, G_i_ and G_q_ G proteins and modulates their signaling by adenylate cyclase activity and generating diacylglycerol and inositol 1,4,5-triphosphate through phospholipase C stimulation (Shryock and Belardinelli, 1997). Expression of all five adrenergic receptors has been investigated in the different parts of AD patients and the expression of α_2_ receptors in particular was markedly increased in the cerebral cortex (Russo-Neustadt and Cotman, 1997). However, to investigate the link between microglia, adrenergic receptors and neuroinflammation in AD several studies have been conducted. Injection of a selective neurotoxin, N-(2-chloroethyl)-N-ethyl-2 bromobenzylamine (DSP4), into a mice model of AD has been demonstrated to increase iNOS and IL-β levels in microglia in Aβ-treated rats. Reversal of iNOS and IL-6 concentration elevations has been observed with co-administration of noradrenalin or isoproterenol, an adrenergic receptor agonist, proving that loss of noradrenaline is connected to Aβ-induced neuroinflammation and consequent death in an animal model of AD (Heneka et al., 2002). In another study, α_2_ levels were found to be significantly increased in the different locations of the brain in AD patients, such as prefrontal cortex and hippocampus cerebral microvessels (Kalaria et al., 1989). In recent times, the α_2A_-adrenergic receptor has been reported to modulate a novel role in norepinephrine release and response control by APP. APP has been found to disrupt the recruitment of arrestin 3, which modulates α_2A_-adrenergic endocytosis (Zhang et al., 2017). Moreover, the α_2A_-adrenergic receptor has been reported to play a crucial role in dexmedetomidine (DMED)-induced improvement in systemic inflammation (SI)-induced cognitive dysfunction. The protective effect of DMED against SI-induced microglial hyperactivation, cognitive impairment and hippocampal neuroinflammation was mediated by the α_2A_-AR signaling pathway, and its involvement was confirmed by blocking its activity by nonspecific α_2A_ receptor antagonist, atipamezole, or the specific antagonist of α_2A_-AR, BRL-44408. However, the results showed that DMED treatment is only effective if treated during the SI (Yamanaka et al., 2017). Furthermore, DMED has been reported to play a crucial role in α_2_-adrenoceptor-mediated microglial polarization in 6-hydroxy dopamine (6-OHDA)-treated BV-2 cells. The 6-OHDA treatment can induce polarization of microglia to the M1 state in BV-2 cells, which was prevented by DMED. Pretreatment with DMED seemed to attenuate 6-OHDA-induced release of different proinflammatory markers, such as IL-6, IL-1β and TNF-α as well as expression of IL-10 and IL-13 along with TGF-β2. Furthermore, DMED also suppressed IL-4-mediated microglial polarization and subsequent expression of microglial M2 markers, Arg-1, resistin-like α (Retnla/Fizz1) and chitinase 3-like 3 (Chi3l3/Ym1; Zhang et al., 2017).

The role of β_2_ receptors in the pathogenesis of AD has gained the most interest among all other adrenergic receptors and is also linked with Aβ generation (Ni et al., 2006; Yu et al., 2011). In addition, chronic treatment with β_2_ adrenergic receptor agonist, isoproterenol, has been implicated to increase Aβ concentrations in murine models of AD accumulation through increasing the activity of γ secretase (Ni et al., 2006). Very recently, in a 5XFAD transgenic mouse model of AD, chronic administration of biased and selective β_1_ adrenergic receptor agonist, xamoterol, reduced Aβ toxicity-associated neuroinflammation and tau by reducing microgliosis and astrogliosis markers, allograft inflammatory factor 1 (Iba1) and glial fibrillary acidic protein (GFAP), as well as inflammatory markers, Iba1, CD74, CD14 and TGFβ. In primary microglia cell cultures, xamoterol diminished TNF-α produced by LPS treatment (Ardestani et al., 2017). Lately, a biased and selective β_1_ adrenergic receptor partial agonist, STD-101-D1, has been shown to suppress TNF-α production and LPS-induced neuroinflammation in both primary microglia and C57Bl/6J mice with high brain permeability (Yi et al., 2017).

### Muscarinic Acetylcholine Receptors

Acetylcholine was one of the first discovered neurotransmitters. In the CNS, there are two types of acetylcholine receptors—nicotinic acetylcholine receptors (nAChRs) and muscarinic acetylcholine receptors (mAChRs). Both nAChRs and mAChRs neurotransmitters mediate neurotransmission by acetylcholine and are involved in learning and cognition (Zhao et al., 2016). These two receptors are widely expressed in neurons and glial cells, whereas expression of mAChrRs on microglia deserves more investigation (Wessler et al., 1998; Ragheb et al., 2001). Further, most of the therapeutic drugs approved by FDA for the symptomatic treatment of AD belongs to the class of AChEI (Clark and Karlawish, 2003). mAChRs are one of the subfamilies of GPCRs involved in many fundamental neurological functions (Kruse et al., 2014). There are five subtypes of mAChRs (M1–M5) reported thus far. They have been implicated in many CNS disorders, including AD (Bonner et al., 1987; Jiang et al., 2014). Based on many expert opinions, M1 mAChRs has been suggested as a potential drug target for AD (Langmead et al., 2008; Melancon et al., 2013). It has been claimed that the G-protein coupling of mAChR M1 in the neocortex of AD patients is connected with cognition, short-term memory, and memory consolidation (Anagnostaras et al., 2003; Tsang et al., 2006). Jiang et al. (2014) verified the uncoupling the mAChR M1/G-proteins as one of the causes of cognitive impairment in AD. Therefore, M1 can be a potential target to develop therapeutic interventions to remedy cognitive deficits. Moreover, it has been described that human microglial mAChR responses to cholinergic agonists induce rapid changes based on activation of Ca^2+^, which acts as a secondary messenger initiating a downstream cascade of signaling pathways in CNS disorders (Zhang et al., 1998; Hasselmo, 2006). Unfortunately, responsiveness to ACh by different microglial mAChRs in various locations of brain has not been broadly studied nor received much attention in the literature (Zhang et al., 1998; Hirayama and Kuriyama, 2001; Nyakas et al., 2011; Lee et al., 2014). Despite many reports surrounding microglial mAChRs in different CNS disorders, there are very few studies related to mAChR-mediated microglial activation in AD (Zhang et al., 1998; Kondo et al., 1999; Gnatek et al., 2012; Shin et al., 2015). Recently, Pannell et al. (2016) studied the functional expression of mAChR in microglia isolated from a mouse model of AD. They noted that nearly 25% of isolated microglia and 60% from a stroke model responded to carbachol, which is an agonist of mAChRs. The result was obtained through using an anti-M3 antibody in fluorescence-activated cell sorting (FACS) analysis (Pannell et al., 2016). Additionally, Joseph et al. (2006) carried out several experiments regarding the effect of the blueberry extract (BBE) on oxidative stress-mediated signaling on Aβ, which induced a change in cognitive function while aging and in dementia in cultured primary hippocampal neuronal cells (HNC). This suggests that BBE suppresses oxidative stress by lowering pCREB and pPKC γ activated by dopamine in M1-transfected cells (Joseph et al., 2006, 2007). A similar result was described for C-2 ceramide-induced stress signaling in COS-7 cells (Joseph et al., 2010).

Recent evidence shows that there is a relationship between APP and M1 and M2 mAChRs from slices of rat cortex, hippocampus, striatum and cerebellum. M1 mAChR increases APP release and M2 mAChR decreases the formation of APPs *in vitro* (Farber et al., 1995). In another study, an increase in amyloidogenic APP processing was observed in M1 mAChR knock-out mice (Davis et al., 2010). In addition, M1 and M2 mAChR activation was also associated with a rise in α-secretase, which can be antagonized by PKC inhibitors or phorbol esters that will activate PKC and increase sAPP release and P3, and as such, Aβ generation will be diminished (Buxbaum et al., 1992; Hung et al., 1993; Farber et al., 1995). Caccamo et al. (2006) reported that M1 mAChR antagonist, dicyclomine, can upregulate BACE1 levels in ADtg mice, whereas AF267B, an M1 mAChR agonist, remarkably decreases Aβ and tau levels in the hippocampus and cortex region of the brain. The deletion of the M1 mAChR gene has also seemed to markedly elevate Aβ plaque formation. Its expression in neurons is sufficient for non-amyloidogenic APP processing. and nonselective inhibition of mAChRs blocks the beneficial effects of M2 and M4 receptors (Farber et al., 1995; Davis et al., 2010). Although there is much less work on microglial mAChRs related to AD progression, this data offers hints for further evaluation to locate other mAChRs, such as M2 on microglia. Since that discovery, the M1 mAChR agonist exerts the most selective and specific attenuation of the pathological hallmarks of AD, and developing a drug based on selective agonists of M1 mAChR for both neurons and microglia, like Xanomeline, hold possibilities for the treatment of AD (Bodick et al., 1997).

### Cannabinoid Receptors

Cannabinoid receptors are G-protein coupled receptors found ubiquitously throughout the brain and the other parts of the body. Their endogenous ligand is endocannabinoid and external ligand is tetrahydrocannabinol (THC), which both inhibit cAMP accumulation. CB receptor signaling pathways couple with inhibitory G-protein (G_i_), and their binding with G_s_ has also been reported (Howlett et al., 1986; Glass and Felder, 1997). They comprise of three receptors, namely CB1, CB2 and GPR55 receptors. Recent evidences suggest that endocannabinoid system is more complicated and it contains additional receptor (Ryberg et al., 2007). In this regards GPR55, previously an orphan receptor which can also be activated many classical cannabinoids has been added as a third cannabinoid receptor (Moriconi et al., 2010). These receptors mediate many psychoactive effects of cannabinoids (Mackie, 2008). The expression of cannabinoid receptors depends on the microglial activation state (Becher and Antel, 1996). CB1 and CB2 are both expressed on microglia and are especially overexpressed in neuroinflammatory disorders (Ma et al., 2015). Expression of CB1 receptors in human microglia is not well documented (Stella, 2010). In rat microglia, CB1 receptor has been found to inhibit NO release from endotoxin or cytokine-activated cortical microglia cells. Cells treated with both high and low-affinity binding enantiomers of cannabinoid CP ((+)-CP56667 and (−)-CP55940) showed dose-dependent inhibition of NO release by LPS stimulation. This attenuation of NO was reversed by pretreatment with pertussis toxin (Gα_i_/Gα_o_ protein inactivator) or cholera toxin (Gα_s_ activator). The presence of CB_1_ receptors in rat microglia was demonstrated by immunoblot assaying with CB1 receptor amine terminal domain-specific antibody and mutagenic reverse transcription-polymerase chain reaction. Moreover, colocalization in microglia was also confirmed by a microglial marker (Waksman et al., 1999). Besides this, CB1 has been reported to play a neuroprotective role in MPTP-induced neurotoxicity in Parkinson’s disease model. Activation of CB1 receptors showed marked improvement in motor function within animal models and survival rate of DA neurons in both the substantia nigra (SN) and striatum. Supposedly, this result is based on suppression of the production of NADPH oxidase reactive oxygen species by microglia and reduction in proinflammatory cytokines, IL-1β and TNF-α, from activated microglia (Chung et al., 2011).

CB2 receptors are highly expressed in inflammatory and primed macrophages and the functional sates of macrophages are also sensitive to the action of CB2 receptors in response to both endogenous and exogenous ligands (Carlisle et al., 2002). Their activation in microglia modulates cell proliferation and migration while also increasing the accumulation of less harmful microglia at sites of injury while also reducing IL-6, TNF-α and free radical production (Facchinetti et al., 2003; Walter et al., 2003; Carrier et al., 2004; Eljaschewitsch et al., 2006; Bisogno and Di Marzo, 2010). To assess the upregulation of CB2 receptors in activated microglia, microglia cells were cultured with microglial activators, IFN-γ and granulocyte macrophage-colony stimulating factor (GM-CSF). Activated microglia were tested for increases in the expression of CB2 receptors, which was an 8- to 10-fold increase over non-treated microglia cells. In another study, it was seen that activation of microglia by both LPS and IFNγ was attenuated by treatment with a cannabinoid CB2 receptor agonist, AM1241, which shifted the microglial state from M1 to M2. To confirm the role of CB2 receptors on the attenuation of microglial activation, microglial cells were treated with a CB2 receptor antagonist, AM630, or chelerythrine, a PKC inhibitor that completely reversed the effect of the agonist (Ma et al., 2015).

In the brain of AD patients, the expression of cannabinoid receptors has been implicated to provide neuroprotection and prevention of neurodegeneration. The expression of cannabinoid receptors was investigated in the hippocampus and entorhinal cortex sections of postmortem brains of AD patients. The expression of the CB2 receptors in the neurite plaques of microglia and astrocytes was very high and selective, but the expression of CB1 receptors was indifferent (Benito et al., 2003). The expression of CB1 in the postmortem brain samples of various AD patients during the clinical deteriorating stage of AD has been described. CB1 expression was initially higher in the selective hippocampal areas and gradually decreased as AD progressed, clearly indicating the involvement of CB1 receptors during the earlier stages of AD (Manuel et al., 2014). In Aβ_1–40_-induced microglial activation within an animal model featuring AD administration of synthetic cannabinoids (HU-210, WIN55, 212-2 and JWH-133), a selective CB2 agonist inhibited Aβ-induced microglial activity with marked changes in microglial activation markers and cell morphology with a release of pro-inflammatory cytokines (TNF-α; Ramírez et al., 2005). In summary, microglial CB1 and CB2 receptors are able to modulate microglial activation in both *in vitro* and *in vivo* models of AD, which provides a potential avenue to develop and design therapeutic agents for the treatment of AD.

Furthermore, the third cannabinoid receptor GPR55 is a novel cannabinoid receptor highly expressed in various regions of brain, especially in the striatum, hippocampus, hypothalamus, frontal cortex, cerebellum, and brain stem (Ryberg et al., 2007). GPR55 can bind to both cannabinoid compounds, such as abnormal-cannabidiol (Abn-CBD), although its endogenous ligand is lysophophatidylinositol (LPI; Shore and Reggio, 2015). The potential of GPR55 in neurodegenerative disorders has been reported in many studies (Shore and Reggio, 2015; Celorrio et al., 2017; Alavi et al., 2018). It is significantly expressed in both primary and BV-2 microglia cells. Stimulation of LPS in BV-2 exhibits downregulation of GPR55 expression upon treatment with LPI. LPI increases ERK phosphorylation in BV-2 cells after IFN-γ treatment, which suggests the involvement of GPR55 in microglia activation and modulating inflammation (Pietr et al., 2009). In addition, how microglial GPR55 mediates neuroprotection by LPI was demonstrated in rat organotypic hippocampal slice cultures (OHSC). Treatment with LPI protects dentate gyrus granule cells from NMDA-induced lesion. The involvement of GPR55 in neuroprotection was confirmed by the deletion of GPR55 through siRNA (Kallendrusch et al., 2013). Furthermore, GPR55 knockout mice exhibited impaired movement coordination in comparison with GPR55 null mutant mice (Wu et al., 2013). Administration of LPI onto the hippocampus of rats showed a reduction in the capability of spatial navigation strategy and worsened the capability of finding the escape tunnel in the barnes-maze (BM), a test that performed to assess spatial learning and memory (memory to place objects). In contrast, rats receiving CID16020046, a GPR55 antagonist, spent less time in the target zone during the test, suggesting the involvement of hippocampal CA1 GPR55 in spatial learning and memory (Marichal-Cancino et al., 2018). Therefore, all these findings indicate a direct connection of GPR55 to motor function, memory formation, and neuroinflammation, but additional research is merited for its role in microglial activation in neurodegeneration. Uncovering the GPR55 pathway in microglia may provide a better understanding of AD.

### Chemokine Receptors

Chemokine receptors are mostly located on microglial membrane and linked with diverse physiological functions, such as neuronal migration, synaptic activity, cell proliferation and even neuronal death. However, different chemokine receptors are reported to be involved in neurodegenerative diseases (Cartier et al., 2005; Mines et al., 2007). There are almost 50 types of chemokines, 18 of which are signal transducing and five acting as decoy/scavenger receptors. They are further grouped into two major categories (Proudfoot, 2002). Four chemokine receptors, CCR3, CCR5, CXCR2 and CXCR3, and their ligands have been described to be present in the brain of AD patients. Yet, CCR3 and CCR5 are predominantly found in microglia of both control and AD brains, expressing reactive microglia, and MIP-1β expression (ligands for CCR5 and/or CCR3) of reactive astrocytes were seen to be associated with amyloid deposits (Xia et al., 1998). Moreover, with the administration of Aβ peptide in microglia isolated from the brain of AD and control patients, CXCL8, CCL2, and CCL3 levels increased in a dose-dependent manner (Cartier et al., 2005). Another report on microglia isolated from postmortem human brains showed upregulation of CXCL8, CCL2, CCL3 and CCL4 to a lesser extent after incubation with Aβ (Walker et al., 2001). Furthermore, it has been noted by Cardona et al. (2006) that the CCR3, CCR5, CX_3_CR1, CXCR2 and CXCR3 expressed in microglia were associated with senile plaques, whereas expression of CX_3_CR1 was determined as high in microglia and its ligand CX_3_CL1 in neurons. They also reported the involvement of CX_3_CR1 signaling to protect against microglial neurotoxicity in three different *in vivo* models (Hickman and El Khoury, 2010). Besides that, CXCR2 has been investigated in order to establish its involvement in Aβ production and γ-secretase activity. The findings suggest that CXCR2-mediated Aβ production is regulated by diminishing γ-secretase activity (Bakshi et al., 2008). Overall, this evidence supports the idea there is involvement of chemokine receptors in the recruitment and accumulation of microglia in AD. Determining the precise chemokine receptors and understanding the underlying mechanism of chemokine receptor-mediated signaling will aid in develop therapeutic strategies to treat AD.

### G-Protein-Coupled Formyl Peptide Receptor-Like 1 (FPRL1)

G-protein-coupled formyl peptide receptor-like 1 (FPRL1) and its mouse homolog, FPR2, binds Aβ_42_ and activates microglia, maintaining its chemotactic activity in AD (Iribarren et al., 2005). FPR2 binds to Aβ and mediates Aβ uptake in glia-infiltrating senile plaques. To determine the relationship between Aβ and resolution of inflammation, microglia cells were treated with Aβ_42_, and the result showed that FPR2, α7nAChR, and (PPAR)-δ was downregulated by Aβ_42_ treatment, indicating the expression of FPR2 is correlated with resolution of inflammation and dysfunction in AD (Zhu et al., 2015). Annexin A1 (ANXA1), an anti-inflammatory mediator, has been reported to suppress activation of microglial activation through formyl peptide receptor-like 1 (FPRL1/FPR2) signaling (Gavins and Hickey, 2012). In a more contemporary work, ANXA1 was investigated as an anti-inflammatory agent and whether it had any effect on Aβ clearance and degradation in both human patients and a 5XFAD mice model. The result showed that FPR2 levels rose in both human patient and mice models. The Aβ concentration was significantly reduced by enzymatic degradation in N2a cells followed by treatment with ANXA1. It was claimed that this effect was based on the FRP2 receptor (Ries et al., 2016). Humanin, a neuroprotective peptide, has been reported to ameliorate pathological changes and cognitive deficits in AD models induced by Aβ-peptide (Niikura et al., 2011; Chai et al., 2014). In addition, humanin also suppresses Aβ-induced aggregation and fibrillary formation in mononuclear phagocytes. Moreover, humanin is reported to exert neuroprotective effects by inducing chemotaxis of mononuclear phagocytes by both human FPRL1 and its murine counterpart, FPR2 (Ying et al., 2004). In primary mouse microglia cells, N9 treatment and incubation with TNF-α was shown to increase FPR2 gene expression and decrease the chemotactic response against Aβ_42_. Meanwhile, the effect of TNF-α on increasing FRP2 expression was based on MAPK p38 and dependent on the p55 TNF-α receptor (Cui et al., 2002). On the contrary, IL-4 and TGFβ were observed to inhibit mFPR2 in mice (Iribarren et al., 2005). Additionally, TNF-α’s synergistic effect was observed through IL-10 (Iribarren et al., 2007). Palmitoyl-cys[(RS)-2, 3-di(palmitoyloxy)-propyl]-Ala-Gly-OH (PamCAG), a TLR2 muramyl dipeptide (MDP) ligand featuring an intracellular receptor nucleotide-binding oligomerization domain 2 (NOD2) ligand has been reported to upregulate mouse mFPR2 (Chen et al., 2008). With this, receptors for advanced glycation endproducts (RAGE) and both FPR2 and FPRL1 are thought to play important roles in Aβ_1–42_-mediated signal transduction in glial cells. The interaction between RAGE and FPRs are further investigated by co-immunoprecipitation and fluorescence microscopy (Slowik et al., 2012). This finding revealed the missing link and clarifies the explanation of the broad ligand spectrum of FPRs and a clear mechanism underlying their anti-inflammatory and neuroprotective activity. However, to completely understand FPR1/2-mediated chemotactic activity in AD, further investigation is necessary.

### Chemokine-Like Receptor 1 (CMKLR1)

The chemerin receptor chemokine-like receptor 1 (CMKLR1), a homolog of FPR2, is also a functional receptor of Aβ_42_. Although these two receptors bind to the same Aβ, they have a different response. Unlike FPR2, CMKLR1 acts through different pathways, like activation of ERK1/2, PKA, and Akt, and facilitates processing and clearance of Aβ_42_. CMKLR1 also lacks Aβ-induced Ca^2+^ mobilization in CMKLR1-RBL cells (Peng et al., 2015). This lack of secondary messenger is connected with many signaling pathways responsible for downstream activation of GPCRs. Very little is known about microglial CMKLR1 receptors and the pathway associated with its binding with Aβ_42_, further detailed investigation is needed to establish its potential as a therapeutic target for AD.

### Orphan GPCRs

There are almost 140 GPCRs lacking any known endogenous ligands. These GPCRs are thus termed orphan GPCRs. Despite the sophisticated advancement of GPCR-based drug design and discovery, orphan receptors remain less popular therapeutic drug targets (Stockert and Devi, 2015). The orphan GPCR, GPR3, has been reported to be a potential therapeutic target for AD treatment (Ruiz-Medina et al., 2011). Internalized GPR3 with β arrestin 2 promotes Aβ production. Moreover, GPR3-mediated Aβ production is regulated by the cleavage of APP (Nelson and Sheng, 2013). The overexpression of GPR3 in a transgenic mouse model confirmed the claim of the generation of Aβ_42_ and Aβ_40_. The generation of Aβ was based on the cleavage of APP by γ-secretase without any alteration to the expression of γ-secretase subunits. On the other hand, genetic depletion of GPR3 in mice showed a marked decrease in both Aβ_40_ and Aβ_42_ generation (Thathiah et al., 2009; Huang et al., 2015). Although there is a very little or no work of orphan GPCRs in microglial activation, it has been speculated that previously unidentified abnormal cannabidiol (Abn-CBD) receptors, GPR18, is linked with abnormal CBD-mediated microglial migration. In addition, it was noted that microglia abundantly express GPR18 on the cell surface. N-arachidonoyl glycine (NAGly), a GPR18 agonist, also mimics abnormal CBD activity and potentiates microglial migration through MAPK activation in response to CNS damage (McHugh et al., 2010). This exquisite finding suggests the involvement of orphan GPCRs in microglial migration to a pre- or pro-inflammatory state through lipid-based signaling mechanisms in terms of CNS stimuli.

### G-Protein-Coupled Receptor Kinase

G-protein-coupled receptor kinase is responsible for homologous desensitization of retarded GPCRs, prolongation of GPCR activity and is widely assumed to selectively phosphorylate GPCRs (Li et al., 2015). Microglial GRK2 is well-known for its cell-specific regulation severity and transition of pain from acute to chronic (Eijkelkamp et al., 2010; Kavelaars et al., 2011). Suo et al. (2004) documented that at prodromal and earlier stages of the development of AD, Aβ and GPCR kinase-2/5 (GRK2/5) dysfunction at sub-threshold levels is relevant. An *in vitro* study of Aβ treatment noted reduced GRK2/5 levels in the membrane of murine microglia cells, and an *in vivo* study in ADtg mice (CRND8) showed similar results. These findings indicated a novel link between Aβ accumulation and GRK at an early stage of AD development (Suo et al., 2004). Since, Aβ-induced neurotoxicity and inflammation plays a pivotal role in the progression of AD, an in-depth understanding of the role of GRKs in Aβ-induced neurotoxicity may provide critical knowledge of AD pathogenesis.

## Perspective and Conclusions

After decades of research and clinical trials, still, there is no definitive treatment option for AD (Godyn et al., 2016; Hung and Fu, 2017). Seeing the conventional strategy for selecting a therapeutic agent is based on the characteristics of prevention, clearance and degradation of Aβ peptides, these can also be achieved by targeting microglial GPCRs involved in different stages from Aβ generation, degradation, and clearance in the brain. Targeting specific microglial GPCRs at a specific stage of AD progression will not only offer better options for symptomatic treatment, but also a therapeutically potential disease modification. In this article, we have thoroughly discussed GPCR-linked microglial activation in AD, which also sheds light onto the potential of microglial GPCRs for strategic therapeutic intervention for AD. Changes in receptors as well as pre- and pro- inflammatory markers expressed during the various stages of microglia activation are a potential research avenue to completely understand microglial characteristics in many neurodegenerative disorders (Hanisch, 2013). The role of different microglia GPCRs, such as adenosine receptors (A_1_, A_2A_, A_2B_ and A_3_) has been found to be directly linked with microglia activation. Modulation of these receptors has been found to suppress microglia activation and induce Aβ toxicity and neuronal damage. In addition, microglial adrenergic α_2A_ receptors have impact on microglial polarization towards the M1 state. Moreover, both microglial adenosine and adrenergic receptor’s role in Aβ toxicity and cognitive dysfunction show promise. Different microglial GPCRs are linked with Aβ generation. Microglial β_2_ adrenergic receptor and mAChRs, especially M1 have distinct roles in Aβ generation. Activation of microglial M1 mAChR increases α-secretase and sAPP production. Although the presence of M2 and M4 subtypes on microglia requires further evidence, the M1 receptor acts in concert with these two mAChR subtypes (Farber et al., 1995). The microglial mGluRs are also involved in Aβ generation through modulating non-amyloidogenic cleavage of APP and sAPPα activity. Group II microglial mGluR activation can elevate sAPPα and decrease Aβ concentrations. They also promote non-amyloidogenic cleavage of APP, thus decreasing amyloid levels in AD brain. Besides the role of GPCRs in microglial activation, polarization and migration towards neuronal damage, degradation of Aβ and microglia-mediated neurotoxicity is prominently featured in Aβ-mediated neurotoxicity. Microglial P2 (described earlier) and α7 nAChRs have been found to play a crucial role in Aβ degradation and clearance through microglial phagocytosis and chemotaxis. Similarly, two microglial Aβ receptors, FPRL1 and CMKLR1, are responsible for microglial chemotaxis and microglia-mediated Aβ degradation and clearance. Very recently, the orphan receptor, GPR18, has been reported to promote microglial migration towards neuronal damage, though more experimental support is necessary to clarify its role in AD progression. Furthermore, a few GPCRs are related to microglia-mediated neurotoxicity and microglia-neuronal bidirectional communication, which plays a vital part in AD pathology, such as mGluRs. At present, the involvement of microglial cannabinoid receptors and chemokine receptors are gaining much attention regarding Aβ toxicity and Aβ-induced microglial activity. Therefore, targeting cannabinoid receptors and chemokine receptors may serve as additional promising AD therapeutic targets in the future.

Generally, symptoms associated with AD are ambiguous in pathological origin and also linked with different individual GPCRs. Aβ can bind to a single microglial GPCR or with other co-receptors. Depending on the binding of Aβ to different microglial GPCRs GPCR-Aβ-mediated signaling cascade may vary. Therefore, knowledge of these co-receptors or receptor complexes is a prerequisite to understanding Aβ-mediated neurotoxicity. Therefore, targeting more than one GPCR or GPCR with other receptors involved in AD pathogenicity can be a strategic approach to design and develop new therapeutic agents that can counter the multifaceted pathogenesis of AD. For the better understanding of AD pathophysiology, more advanced and sophisticated animal models are required to mimic specific pathologic conditions for AD, such as specific GPCR knockout mice, different microglial cells representing various activated phenotypes for specific targets and incorporating different gene editing tools may also prove effective. Microglia are generally restless—they frequently change their phenotype. Recently, it has been speculated that microglia may show heterogenicity in their activity depending on their location in the brain and in accordance with the type of stimuli (Town et al., 2005; Arcuri et al., 2017).

The success rate of GPCR-targeted therapeutic agents in 2013–2017 reported by FDA in phases I, II and III in terms of clinical trials are 78%, 39% and 29%, respectively. This is comparatively higher than the FDA’s average for other drug candidates currently on trial (Hauser et al., 2017). The major limitation of developing therapeutic agents for AD is the lack of mechanistic selection criteria for drug targets based on biological markers that can meet clinical efficacy standards. To do so, classification and selection of GPCRs based on functional activity rather than conventional structural similarity will reduce the burden of screening and designing GPCR selective drugs. In addition, to identify novel ligands for GPCRs, DNA-encoded libraries may be useful. Potential candidates for GPCRs and their chemical and biological data can be found from reported research and evaluation. However, the chemical and biological data of potential GPCR candidates are not well-organized in online libraries and databases. Yet, this data can be organized and made available through online libraries, such as GPCRdb and CheMBL to facilitate GPCR-based ligand design. A very efficient concept of biased ligand can also be adopted. Biased ligands can stabilize the subsets of receptor conformation to produce novel therapeutic outcomes. The increasing popularity of this ligand concept lies in its capability for designing more safe, tolerable drugs with high efficacy (Violin et al., 2014). Another concept is allosteric modulators—they can remotely modulate receptor activity by endogenous or physiological ligands that induce activity at the binding site. This novel and attractive mechanism can offers superior target selectivity over conventional modulators of GPCRs (Hauser et al., 2017). Another concern regarding targeting GPCRs on microglia is heteromerization of receptors. Heteromerization of receptors is associated with crosstalk between two extracellular signals. Recently, it has been established that appropriate heteromers of GPCRs are important and modification of the pharmacology and signaling properties of drugs depends of selection of unique GPCR heteromers (Albizu et al., 2010). The presence of heterodimers or oligomeric complexes of GPCRs on different cells is now widely known and accepted (Albizu et al., 2010). Although heterodimerization may have implications in the pharmacology of therapeutic agents, the knowledge of regarding the molecular basis of such heteromerization still remains in its initial stage (Milligan, 2004). However, several heteromers of various GPCRs on different tissues have been reported to possess therapeutic relevance to drug design. Co-internalization or coexpression of different receptors, such as α_1a_/α_1b_ adrenoceptors, A_1_/A_2A_, A_1_/mGlu_1α_ and A_2_/mGlu5, CB1/A_2A_ in different types of cells has been reported in both *in vitro* and *in vivo* studies related to a variety of neurological disorders (Ciruela et al., 2001, 2006; Stanasila et al., 2003; Kachroo et al., 2005; Carriba et al., 2007; Tebano et al., 2009). Many drugs possess unique pharmacology and affinity towards GPCR heterodimers, such as anti-parkinsonian drugs featuring more affinity to D_3_/D_2_ than its homodimers (Yoshioka et al., 2001; Maggio et al., 2003). These findings suggest that heterodimers are worthy of more attention as drug targets for neurological disorders. Meanwhile, there is still lack of studies on microglial GPCR heteromerization related to Aβ generation, clearance, and degradation, which should be a prime concern for developing bivalent ligands, such as agonist-agonist, agonist-antagonist or one linked with amino acid spacers. These bivalent ligands can target multiple heteromers at a time and have superior efficacy than single ligand-based therapy (Zhang et al., 2007). Therefore, biased ligands and allosteric modulators, both novel concepts, offer a potential strategy to design GPCR-based drugs for AD.

## Author Contributions

MEH, D-KC and I-SK conceptualized and designed the study. D-KC also supervised and corresponded. MEH reviewed the literature and wrote the manuscript, drew the figures and made the tables. MJ and MA contributed to the arrangement of data. I-SK contributed to drawing the figures and helped in the revision of the article. All authors read and approved the final manuscript.

## Conflict of Interest Statement

The authors declare that the research was conducted in the absence of any commercial or financial relationships that could be construed as a potential conflict of interest.

## Abbreviations

6-OHDA: 6-hydroxy dopamine
Aβ: Amyloid-β peptide
Abn-CBD: abnormal cannabidiol
AChE: Acetylcholinesterase
AChEI: Acetylcholinesterase inhibitors
AD: Alzheimer disease
APL: Allosterically potentiating ligand
APP: Amyloid-β precursor protein
Arg1: Arginase 1
β-APP: Amyloid-β precursor protein
BACE1: β-site amyloid precursor protein cleaving enzyme 1
CCL2: Chemokine (C-C motif)
CD16/32: Cluster of differentiation 16/32
CGA: Chromogranin A
CNS: Central nervous system
CREB: cAMP response element-binding protein
CXCL10: C-X-C motif chemokine 10
DMED: Dexmedetomidine
FACS: Fluorescence-activated cell sorting
FDA: Food and Drug Administration
FPRL1: G-protein-coupled formyl peptide receptor-like 1
GFAP: Glial fibrillary acidic protein
GPR3: G-Protein-Coupled Receptor 3
GRK: G-protein-coupled receptor kinase
hAPP: Human beta-amyloid precursor protein
hPS1: Human presenilin-1
Iba1: Allograft inflammatory factor 1
IL-1Ra: IL-1 receptor antagonist
LPS: Lipopolysaccharide
mAChRs: muscarinic acetylcholine receptor
MAPK: Mitogen-activated protein kinase
mGluRs: metabotropic glutamate receptors
nAChRs: nicotinic acetylcholine receptor
NAGly: N-arachidonoyl glycine
NFT: Immunoreactive neurofibrillary tangles
PI3 kinase/Akt: phosphatidylinositol 3-kinase/Akt
RAGE: Receptor for advanced glycation endproducts
sAPP: Soluble amyloid Precursor Protein
SR-A1: Scavenger receptor class A1
SR-B1: Scavenger receptor class B1
TGF-β: Transforming growth factor-β
TREM-2: Triggering receptor also expressed on myeloid cells-2
UDP: Uridine 5’-diphosphate
Ym1: Chitinase-like protein 1.
